# shRNA‐mediated PPARα knockdown in human glioma stem cells reduces *in vitro* proliferation and inhibits orthotopic xenograft tumour growth

**DOI:** 10.1002/path.5201

**Published:** 2018-12-27

**Authors:** Harry R Haynes, Helen L Scott, Clare L Killick‐Cole, Gary Shaw, Tim Brend, Kelly M Hares, Juliana Redondo, Kevin C Kemp, Lorena S Ballesteros, Andrew Herman, Oscar Cordero‐Llana, William G Singleton, Francesca Mills, Tom Batstone, Harry Bulstrode, Risto A Kauppinen, Heiko Wurdak, James B Uney, Susan C Short, Alastair Wilkins, Kathreena M Kurian

**Affiliations:** ^1^ Brain Tumour Research Group, Translational Health Sciences Bristol Medical School, University of Bristol Bristol UK; ^2^ Department of Cellular Pathology North Bristol NHS Trust Bristol UK; ^3^ Translational Health Sciences Bristol Medical School, University of Bristol Bristol UK; ^4^ Functional Neurosurgery Research Group, Translational Health Sciences Bristol Medical School, University of Bristol Bristol UK; ^5^ Leeds Institute of Cancer and Pathology University of Leeds Leeds UK; ^6^ Multiple Sclerosis and Stem Cell Group, Translational Health Sciences Bristol Medical School, University of Bristol Bristol UK; ^7^ Flow Cytometry Facility, School of Cellular and Molecular Medicine University of Bristol Bristol UK; ^8^ Department of Neurosurgery North Bristol NHS Trust Bristol UK; ^9^ Department of Clinical Biochemistry North Bristol NHS Trust Bristol UK; ^10^ Bioinformatics Facility, School of Biological Sciences University of Bristol Bristol UK; ^11^ Department of Clinical Neuroscience and Stem Cell Institute University of Cambridge Cambridge UK; ^12^ Clinical Research and Imaging Centre University of Bristol Bristol UK; ^13^ Stem Cells and Brain Tumour Group, Leeds Institute of Cancer and Pathology University of Leeds Leeds UK

**Keywords:** PPARα, glioma stem cell, shRNA

## Abstract

The overall survival for patients with primary glioblastoma is very poor. Glioblastoma contains a subpopulation of glioma stem cells (GSC) that are responsible for tumour initiation, treatment resistance and recurrence. PPARα is a transcription factor involved in the control of lipid, carbohydrate and amino acid metabolism. We have recently shown that PPARα gene and protein expression is increased in glioblastoma and has independent clinical prognostic significance in multivariate analyses. In this work, we report that PPARα is overexpressed in GSC compared to foetal neural stem cells. To investigate the role of PPARα in GSC, we knocked down its expression using lentiviral transduction with short hairpin RNA (shRNA). Transduced GSC were tagged with luciferase and stereotactically xenografted into the striatum of NOD‐SCID mice. Bioluminescent and magnetic resonance imaging showed that knockdown (KD) of PPARα reduced the tumourigenicity of GSC *in vivo*. PPARα‐expressing control GSC xenografts formed invasive histological phenocopies of human glioblastoma, whereas PPARα KD GSC xenografts failed to establish viable intracranial tumours. PPARα KD GSC showed significantly reduced proliferative capacity and clonogenic potential *in vitro* with an increase in cellular senescence. In addition, PPARα KD resulted in significant downregulation of the stem cell factors c‐Myc, nestin and SOX2. This was accompanied by downregulation of the PPARα‐target genes and key regulators of fatty acid oxygenation *ACOX1* and *CPT1A*, with no compensatory increase in glycolytic flux. These data establish the aberrant overexpression of PPARα in GSC and demonstrate that this expression functions as an important regulator of tumourigenesis, linking self‐renewal and the malignant phenotype in this aggressive cancer stem cell subpopulation. We conclude that targeting GSC PPARα expression may be a therapeutically beneficial strategy with translational potential as an adjuvant treatment. © 2018 The Authors. *The Journal of Pathology* published by John Wiley & Sons Ltd on behalf of Pathological Society of Great Britain and Ireland.

## Introduction

Gliomas form the most common group of primary central nervous system (CNS) tumours, with an incidence of 6.6 per 100 000 individuals/year 1. A total of 50% of adult gliomas are glioblastomas, which are associated with poor clinical survival 2, 3. The median survival is 15 months in the setting of a clinical trial 4, 5 and 12 months using current treatment regimens 1, 6, 7.

The peroxisome proliferator‐activated receptors (PPARs) are ligand‐activated transcription factors with diverse metabolic functions 8. PPARα activates mitochondrial and peroxisomal fatty acid oxidation and ketogenesis and inhibits glycolysis and fatty acid synthesis 9, 10, 11. Our previous work has shown that the *PPARA* gene and its protein product are significantly overexpressed in IDH‐wild type primary glioblastomas and that high *PPARA* expression functions as an independent prognostic biomarker 12. This finding has been independently cross‐validated in the Chinese Glioma Genome Atlas 13.

PPARα agonists such as fenofibrate have clinical utility in treating dyslipidaemia 14. Fenofibrate reduces glioma cell motility 15, 16 and induces cell cycle arrest and apoptosis *in vitro* 17, 18. Fenofibrate has also been reported to exert anti‐tumour effects by inducing ketogenesis 19 and reducing glycolytic flux 20, 21.

Stem‐like cells have been identified in glioma *in vitro* models 22, 23 and glioma stem cells (GSC), with the defining properties of self‐renewal, multi‐potency and *in vivo* tumourigenicity being isolated from human glioblastoma samples 24, 25, 26. GSC are considered responsible for tumour recurrence and treatment failure 27, 28. Karyotypically normal, untransformed (foetal) neural stem cells (NSC) share many features with patient‐derived GSC 29 and are ideal experimental controls 30. In order to improve our understanding of GSC biology, the key regulatory pathways driving the proliferation of this cancer stem cell population need to be understood. Identification of factors that distinguish NSC from transformed GSC may lead to new therapeutic agents designed to inhibit neoplastic growth with minimal toxicity to the (adult) NSC compartment 31.

Several studies to date suggest that PPARα signalling contributes to the proliferation of glioblastomas 12, 32. However, the role of PPARα expression in human GSC populations is unknown. In this study, we tested the hypothesis that PPARα expression contributes to the malignant phenotype of GSC. We used RNA interference approaches to establish the role of PPARα in maintaining the properties of GSC.

## Methods

### Cell culture

The human GSC (G144 and G26) and NSC (U5 and U3) cell lines (kind gifts from Dr Steve Pollard, University of Edinburgh) were cultured as monolayers in serum‐free basal media 26, 29. HEK293T (human embryonal kidney) cells (Sigma, St. Louis, MO, USA) used for producing lentiviral particles were cultured in DMEM (10% FBS and 1× non‐essential amino acids). All cell lines were cultured in 5% CO_2_ at 37 °C.

### Protein and RNA extraction

Total protein was extracted from cell lines using Milliplex lysis buffer (Millipore, Burlington, MA, USA) and quantified using a Qubit^®^ Protein kit and fluorometer (Life Technologies, Carlsbad, CA, USA). RNA was extracted using an RNeasy^®^ Plus Mini Kit (Qiagen, Hilden, Germany) and the QIAcube^®^ platform. RNA was quantified using a NanoDrop1000 spectrophotometer (ThermoFisher Scientific, Waltham, MA, USA).

### Analysis of GSC and NSC accessioned microarray data

Array data derived by Pollard *et al* (GSE15209) 26 was accessed from https://www.ncbi.nlm.nih.gov/geo/query/acc.cgi. Data analysis was performed using Partek Genomics Suite v.6.16.0812 (Partek, St. Louis, MO, USA) and normalised using GC‐RM*A*. Differentially expressed genes were analysed using an ANOVA. The false discovery rate was set at an FDR‐corrected *p* value of <0.05 with a 1.5‐fold expression change cut‐off.

### *PPARA* shRNA oligonucleotide design

Human (NCBI Gene ID: 5465) *PPARA (*22q13) shRNA sequence primers were designed as previously described 33 using the BLOCK‐iT™ RNAi Designer (https://rnaidesigner.thermofisher.com/rnaiexpress/). Double‐stranded shRNA constructs with an upstream U6 promoter were produced using a pSilencer plasmid as the PCR template. The forward primer sequence was 5′‐CGACTCACTATAGGGCGAATTGGGT‐3′, and the reverse primer sequence contained the shRNA oligonucleotide added to the 5′ tail (see supplementary material, Table S1). Cloning of shRNA expression cassettes was carried out as previously described 33, 34, and the resulting shRNA plasmids were validated using Sanger sequencing (SourceBioscience; Nottingham, UK).

### Generation of recombinant lentiviral particles and transduction

U6.shRNA and scramble (SCR) cassettes were cloned into an EGFP‐expressing lentiviral backbone (pRRL.sin.cppt.CMV.EGFP.WPRE). Viral particles were produced and titred as described previously 35. Concentrated lentiviral particles were added to G26 cells for 72 h (multiplicity of infection = 20). Stable PPARα protein knockdown (KD) was established, and a luciferase‐expressing cassette (pCignal Lenti‐TRE‐Reporter, CLS‐PCR‐1, Qiagen) was transduced into the cells using polybrene (Sigma) at 8 μg/ml before puromycin selection.

### *In vitro* cell proliferation studies

Cells were plated at 420 cells/mm^2^ and cultured for 72 h. The total cell number for each replicate for each line was counted. Cells were re‐plated at 420 cells/mm^2^, and the experiment was repeated every 72 h for 15 days. The fold increase in cell number over day 0 was calculated using the mean value of each technical replicate for each cell line at each independent time point. Ki67 and caspase‐3 fluorescence immunocytochemistry was carried out as described previously 36 using antibodies listed in supplementary material, Supplementary materials and methods. CellTrace™ Violet proliferation studies were carried out according to the manufacturer's instructions (Thermofisher). The proliferation control and experimental samples were acquired on a Novocyte 3000 Flow Cytometer (Acea Biosciences, San Diego, CA, USA). Data were analysed using ModFit LT v3.3 software (Verity Software House, Topsham, ME, USA). Cell cycle analysis was carried out on the platforms described above using 5 μm Draq5 nuclear stain (BioLegend, San Diego, CA, USA) (15 min incubation) and cells fixed in 4% paraformaldehyde (PFA).

### Colony‐forming unit assay

Cells were plated at 16 cells/mm^2^ and cultured for 12 days. The cells were fixed (4% PFA) and then stained with 1% crystal violet (Sigma). Calculation of colony‐forming unit (CFU) efficiency was determined as described previously 37.

### Senescence‐associated β‐galactosidase staining

Cells were plated at 520 cells/mm^2^ and cultured for 5 days. Cells were stained for 12 h using a Senescence β‐galactosidase Staining Kit (Cell Signalling Technologies, Danvers, MA, USA). Ten high‐power fields (hpf) were examined per well and positive (cytoplasmic and nuclear blue) staining recorded as a percentage of total live cells per hpf.

### Intracranial xenografting procedure, bioluminescent imagining and MRI

All animal‐handling procedures and experiments were performed in accordance with the UK Animal Scientific Procedures Act 1986 and covered by UK Home Office licenses (University of Leeds ethics committee project license:PA5C8BDBF).

KD and SCR stably transduced cells were injected into 7‐week‐old female NOD‐SCID (NOD.CB17‐Prkdc^scid^/NcrCrl) mice (Charles River, Wilmington, MA, USA); 30 000 cells were engrafted per animal (10 animals per cell line). Intracranial injection co‐ordinates were 1 mm rostral to bregma, 1.5 mm lateral (right) and 4 mm deep. Intracranial tumour growth was analysed every 30 days using the Xenogen IVIS Spectrum *in vivo* imaging system and 60 mg/kg intraperitoneal d‐luciferin (Perkin Elmer, Waltham, MA, USA). MRI data were acquired using a 7 T MRI System (AspectImaging, Watford, UK). NIfTI format images were analysed using MANGO (Mango Software, University of Texas, TX, USA). Animals that had lost ≥20% of body weight or showed persistent neurological signs were terminated by pentobarbitone overdose followed by transcardial 4% PFA perfusion. The brain was removed and fixed in 4% PFA. The experiment ran for 25 weeks.

### Immunohistochemistry (IHC) and immunofluorescence (IF)

Murine brain tissue was processed on a Leica Peloris II histological platform (Leica, Wetzlar, Germany) and H&E stained using a Leica Autostainer XL platform (Leica). PPARα, Ki67 and EGFR IHC was carried out using a Leica Bond III automated immunostainer (Leica). IDH1, ATRX and GFAP IHC were carried out using a Ventana BenchMark ULTRA platform (Roche, Basel, Switzerland). Antigen retrieval techniques and antibody concentrations are detailed in supplementary material, Supplementary materials and methods and Table S4. EGFP immunofluorescence was carried out as described previously 38.

### Western blotting and RT‐qPCR (reverse transcription‐quantitative PCR)

Western blotting was carried out as described previously 36 (primary antibodies are listed in supplementary material, Table S2). Extracted total RNA was reverse transcribed to cDNA for quantitative real‐time PCR using a High Capacity cDNA Reverse Transcription Kit (Applied Biosystems, Foster City, CA, USA); qPCR was performed using a StepOne Plus Real‐Time PCR system and StepOne software v2.1 (Applied Biosystems) with Taqman^®^ Fast Gene Expression Mastermix (Applied Biosystems), and Assay On Demand (AOD) products as listed in supplementary material, Table S3.

### Lactate and glucose assays

Cells were plated at 1000 cells/mm^2^ and cultured for 72–96 h. Adherent cells were counted, and the culture media was collected, centrifuged at 160 × *g* and the supernatant kept on ice. Lactate and glucose supernatant concentrations were determined using a Cobas 8000 automated analyser (Roche) (lactate oxidase and hexokinase methods, respectively).

### Statistical analysis

The normality of data distributions were tested using the Kolmogorov–Smirnov and D'Agostino and Pearson tests. A Wilcoxon matched pairs test or unpaired *t*‐test was used as appropriate. A Friedman test with Dunn's multiple comparison test was used for paired non‐parametric analysis of greater than two groups. A two‐way repeated‐measures ANOVA was used to compare *in vitro* cellular growth rates. All statistical tests were two‐tailed. Differences with *p* < 0.05 were considered statistically significant. Data are represented as mean ± SEM (geometric mean ± 95% CI for RT‐qPCR data). Statistical tests were performed using GraphPad Prism v5 (GraphPad Inc., San Diego, CA, USA).

## Results

### PPARα protein and *PPARA* mRNA levels were greater in GSC

**Figure 1:**
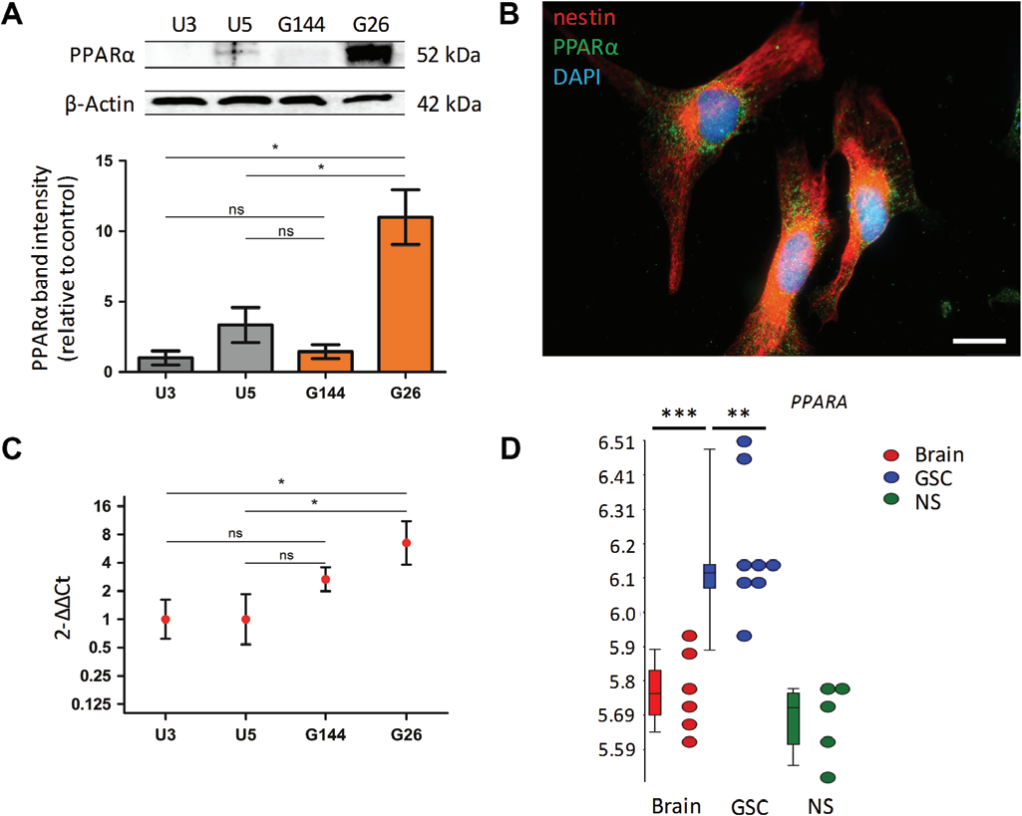
PPARα protein and *PPARA* gene expression are increased in GSC. (A) PPARα protein expression was examined in two NSC lines and two GSC lines at three independent passages, *n* = 3. Protein expression values determined using densitometric analysis, with PPARα‐integrated area density values expressed relative to the loading control β‐actin values. Expression values were calculated relative to the grouped U3 control protein homogenates. Results of equivalent statistical significance were obtained when expression values were calculated relative to the grouped U5 control protein homogenates. (B) High‐power immunofluorescence microscopy showing mixed nuclear/perinuclear and cytoplasmic expression of PPARα; ×630: oil immersion. Scale bar = 25 μm. (C) *PPARA* mRNA expression was examined in NSC control and GSC *in vitro* models by RT‐qPCR, normalised to the reference genes *18S* and *GAPDH* (not shown). Expression values were calculated relative to the grouped U3 control samples. Results of equivalent statistical significance were obtained when expression values were calculated relative to the grouped U5 control samples. The geometric mean and 95% confidence interval are shown on a logarithmic scale (to base2). *n* = 3 independent experiments, all samples analysed in triplicate. (D) *PPARA* expression in GSC (G166, G174, G179, G144, GliNS) versus NSC versus normal adult brain tissue. In the box plots, the upper and lower ‘hinges’ correspond to the 25th and 75th percentiles, respectively. The upper/lower whisker extends to the highest/lowest value that is within 1.5× interquartile range (IQR). Data beyond the end of the whiskers are outliers. Normalised and log‐transformed mRNA gene‐level summaries are shown. The test statistic was a Friedman test with Dunn's multiple comparison test (A and C) or a one‐way ANOVA (D). Error bars show SEM. *p < 0.05, ** *p* < 0.01; ***p < 0.001; ns, non‐significant; GAPDH, glyceraldehyde‐3‐phosphate dehydrogenase; 18S, 18 S ribosomal RNA.

PPARα protein expression was examined in three independent passages of the U3 and U5 NSC lines and G144 and G26 GSC lines. There was a significant increase in PPARα protein level in the G26 cell line compared to both U3 (*p* = 0.032) and U5 cell lines (*p* = 0.048) (Figure 1A). Immunofluorescence microscopy showed a mixed nuclear/perinuclear and cytoplasmic expression of PPARα in the GSC (Figure 1B). RT‐qPCR was performed for the U3, U5, G144 and G26 cell lines: there was a significant increase in *PPARA* mRNA levels in the G26 cell line compared to the U3 cell line (*p* = 0.039) and the U5 cell line (*p* = 0.049) when normalised to *GAPDH* or *18S* expression (Figure 1C).

### *PPARA* gene expression was increased in whole transcriptome analysis of GSC versus NSC

Whole transcriptome expression profile data (accession number GSE15209) were analysed. Using a 1.5‐fold change cut‐off value (FDR threshold of 0.05), analysis of *PPARA* expression showed that this transcript was significantly increased in GSC compared to NSC and normal adult brain tissue (*p* = 0.006, *p* = 0.001, respectively) (Figure 1D). Increased expression of *PPARA* was noted to be within the second quintile of all overexpressed transcripts within the GSC versus NSC comparison (*p* = 0.006, 1.65‐fold change).

### PPARα KD inhibited GSC proliferation and clonogenicity *in vitro*

To investigate the role of PPARα expression in GSC, we generated a stable PPARα KD GSC cell line from the G26 parent line. A control scrambled (SCR) shRNA lentiviral construct was utilised. shRNA‐mediated KD of PPARα was confirmed by western blotting 60 days after lentiviral transduction (see supplementary material, Figure S1). The addition of a luciferase cassette had no effect on shRNA PPARα KD efficiency.

**Figure 2:**
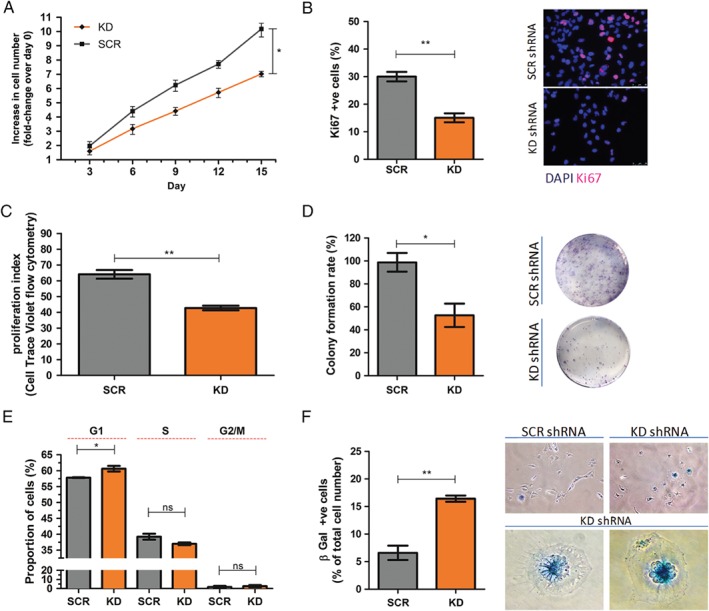
PPARα KD inhibited GSC proliferation and clonogenicity *in vitro*. (A) There was a significant fold decrease in proliferation in the PPARα KD GSC population compared to the SCR shRNA GSC population (population doubling time: 2.3 days versus 1.3 days, KD shRNA and SCR shRNA, respectively). The test statistic was a two‐way repeated‐measures ANOVA and Bonferroni *post hoc* test. Data were analysed using nonlinear regression with *y* = 0 (constrained). Increase (fold‐change) in cell number shown on a logarithmic scale (to base2). (B) There was a significant reduction of the Ki67 index in the PPARα KD GSC population compared to the SCR shRNA GSC population. The proportion of Ki67 nuclear positivity was quantified as the proportion of total nuclei per high‐power field (×200). Ten high‐power fields were examined per slide/technical replicate. Nuclei labelled with DAPI nuclear dye. *n* = 3, three technical replicates per independent experiment. Representative Ki67 IF images shown. Scale bar = 50 μm. (C) CTV cell proliferation assay; PPARα KD GSC showed a reduction in proliferation index (sum of the cells in all generations divided by the computed number of original parent cells theoretically present at the start of the experiment, where each daughter cell has half the CTV fluorescence intensity of its parental cell). Analysis was carried out using a Novocyte 3000 Flow Cytometer with 405 nm excitation laser and 445/45 nm Band Pass (BP) filter. *n* = 3, three technical replicates per independent experiment. (D) There was a significant increase in G1 phase cells with PPARα KD. Draq5 analysis was carried out using a Novocyte 3000 Flow Cytometer with 640 nm excitation laser and 780/60 nm BP filter. *n* = 3, three technical replicates per independent experiment. (E) There was a significant reduction in the number of colonies in the PPARα KD GSC population compared to the SCR shRNA cell population. Representative images of clonogenic assays are shown. (F) There was a significant increase in senescence‐associated β‐galactosidase staining in the PPARα KD GSC population compared to the SCR shRNA cell population. Representative high‐power images of β‐galactosidase staining are shown. *n* = 3, three technical replicates per independent passage. The test statistic was a Wilcoxon matched pair test, two‐tailed *p* value (B–F). Error bars show SEM. SCR, scrambled control; *p < 0.05, **p < 0.01.

PPARα KD lead to a significant decrease in the PPARα KD cell population expansion compared to the SCR shRNA cell population (*p* = 0.021) (population doubling time: 2.3 days versus 1.3 days for KD shRNA and SCR shRNA, respectively) (Figure 2A). There was a significant decrease in Ki67 nuclear positivity between SCR shRNA‐ versus PPARα KD shRNA‐transduced cells (30.0% versus 15.1%) (*p* = 0.003) (Figure 2B).

A CellTrace Violet (CTV) cell proliferation assay was used to monitor cell divisions (generations) in PPARα KD shRNA‐ and SCR shRNA‐transduced cells. In keeping with the population doubling studies described above, PPARα KD shRNA‐transduced cells showed a significant reduction in proliferation index compared to SCR shRNA‐transduced cells (*p* = 0.002) (Figure 2C). In addition, the proportion of cells in the G1 phase of the cell cycle was shown to be significantly increased in the PPARα KD shRNA cell line compared to the SCR shRNA cell line (*p* = 0.034) (Figure 2D). We also studied the effect of stable PPARα KD on clonogenicity. The mean number of colonies formed by PPARα KD cells was reduced by 53.5% relative to SCR shRNA cells (*p* = 0.029) (Figure 2E). There was also a significant increase in β‐galactosidase (pH 6.0) positivity, a known characteristic of senescent cells, between SCR shRNA‐ versus PPARα KD shRNA‐transduced cells (6.8% versus 16.4%, *p* = 0.008) (Figure 2F). In conjunction with this, PPARα KD shRNA‐transduced cells were found to have aberrant cytonuclear features compared to SCR shRNA controls: the cells were notably larger and flattened with a frequent loss of the spindle morphology. Increased intracytoplasmic vacuolation and multi‐nucleation was also noted with strong perinuclear β‐galactosidase positivity (Figure 2F).

### PPARα kD suppressed the tumourigenicity of GSC orthotopic xenografts

**Figure 3:**
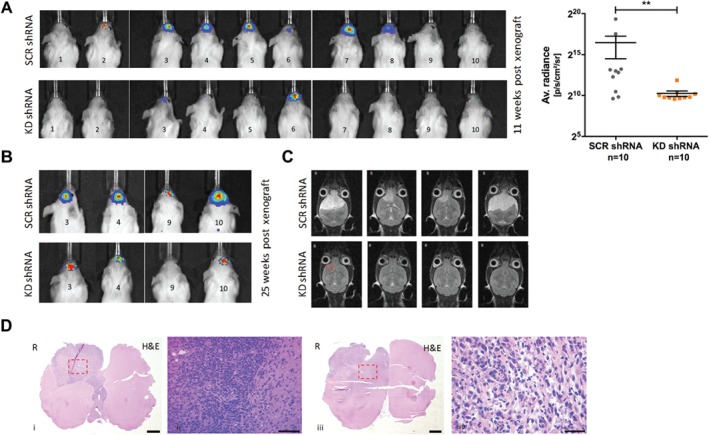
PPARα KD inhibited GSC tumourigenicity *in vivo*. (A) SCR‐ and KD shRNA‐transduced G26 cells were stereotactically implanted in a NOD‐SCID orthotopic murine model and the effect on tumour initiation and progression monitored. At 11 weeks after the xenografting procedure, all animals showed detectable BLI signal (p/s/cm^2^/sr). There was a significant decrease in BLI signal in the PPARα KD group compared to the SCR shRNA control group. (B) All remaining animals (*n* = 8) were terminated after 25 weeks. (C) T2‐weighted MRI was performed 2 h antemortem. The SCR shRNA group showed evidence of extensive right‐sided hemispheric T2‐hyperintense masses (axial). The PPARα KD experimental group showed no radiological evidence of any intracranial abnormality (red box denotes stereotactic injection site). (D) A total of 25 weeks after the xenograft procedure, low and power histological examination of the brains from the control SCR shRNA xenograft arm demonstrated tumour formation. All SCR shRNA xenograft experiments produced tumour masses with histological (H&E) evidence of high cellularity with pleomorphic tumour cells. Representative images shown (coronal sections). Red boxes denote areas shown at greater magnification. Scale bar = 1000 μm (i, iii); scale bar = 100 μm (ii); scale bar = 50 μm (iv). R, right hand side.

SCR and PPARα KD shRNA‐transduced G26 cells were stereotactically implanted in a NOD SCID murine model, and the effect on tumour initiation and progression was monitored. Fourteen days after xenografting, all animals showed detectable bioluminescence (BLI) signal. There was significantly less BLI signal in the PPARα KD group compared to the SCR shRNA control group at each time point during the course of the experiment (Figure 3A). Remaining animals (*n* = 8) were terminated after 25 weeks (Figure 3B). T2‐weighted MRI was performed 2 h antemortem. The SCR shRNA group showed evidence of right‐sided hemispheric T2‐hyperintense lesions with mass effect (Figure 3C). The PPARα KD experimental group showed no MRI signs of intracranial abnormality (Figure 3C). Twenty‐five weeks after the xenograft procedure, low power histological examination of the brains from the control SCR shRNA xenograft arm (*n* = 4) demonstrated extensive tumour formation (Figure 3D).

**Figure 4:**
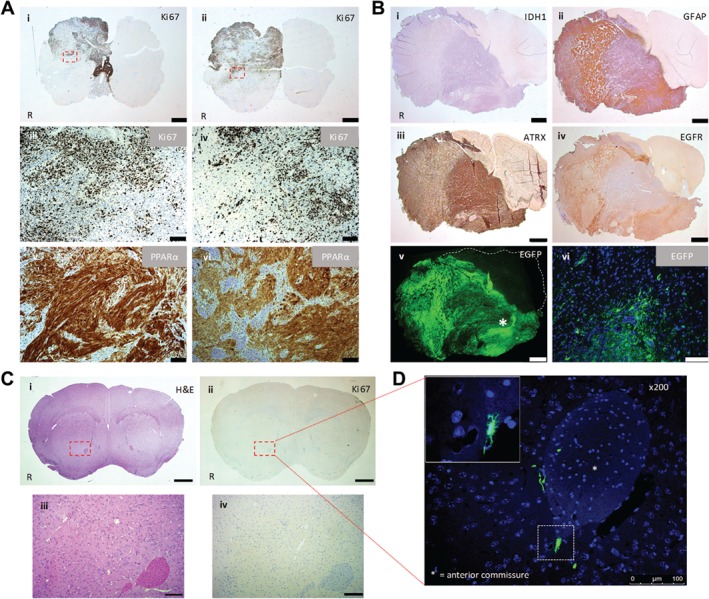
SCR shRNA xenografts: IHC and IF performed on harvested mouse brains 25 weeks after GSC implantation. (A) (i–iv) Representative Ki67 IHC. (v, vi) Representative PPARα IHC. Scale bar = 1000 μm (i, ii); scale bar = 100 μm (iii–vi). (B) SCR shRNA xenograft: IHC and IF (EGFP) performed on harvested mouse brain 25 weeks after GSC implantation. Scale bar = 1000 μm (i–v); scale bar = 50 μm (vi). (C) Representative H&E image of a coronal section and Ki67 IHC performed on a consecutive tissue section. Scale bar = 1000 μm (i, ii); 100 μm (iii, iv). (D) Immunofluorescence microscopy detection of EGFP‐positive cells. The inset in the immunofluorescent image denotes the hatched area ×400. Scale bar = 100 μm. R, right hand side. Red boxes denote areas shown at greater magnification (C and D).

All SCR shRNA xenograft experiments produced tumour masses with histological (H&E) evidence of non‐circumscribed cellular tumours consisting of pleomorphic cells (Figure 3D) with frequent atypical mitotic figures. Ki67 IHC showed variable nuclear positivity across the tumour field (focal areas of >50% Ki67 positivity) and diffuse infiltration by Ki67‐positive cells into the adjacent host parenchyma (Figure 4A). PPARα IHC showed extensive cytoplasmic and nuclear positivity (Figure 4A). IHC performed on SCR shRNA xenografts showed the tumour cells to be negative for the expression of the IDH1R132H‐mutated protein product with strong nuclear ATRX expression and GFAP and EGFR immunopositivity (Figure 4B). EGFP expression examined by immunofluorescence recapitulated the malignant infiltration into the host parenchyma described above (Figure 4B). In contrast, the KD shRNA xenograft arm of the experiment showed no histological evidence of tumour formation (Figure 4C). Immunofluorescence microscopy of brains from the KD shRNA xenograft arm of the experiment demonstrated single cells with EGFP immunopositivity (negative for human‐specific Ki67; Figure 4C). These cells were scattered at the lateral aspect of the right anterior commissure, an area just medial to the stereotactic injection site (Figure 4D). No EGFP‐positive cells were observed in any other brain regions.

### PPARα shRNA KD altered the protein and gene expression of stem cell and mitogenic markers

**Figure 5:**
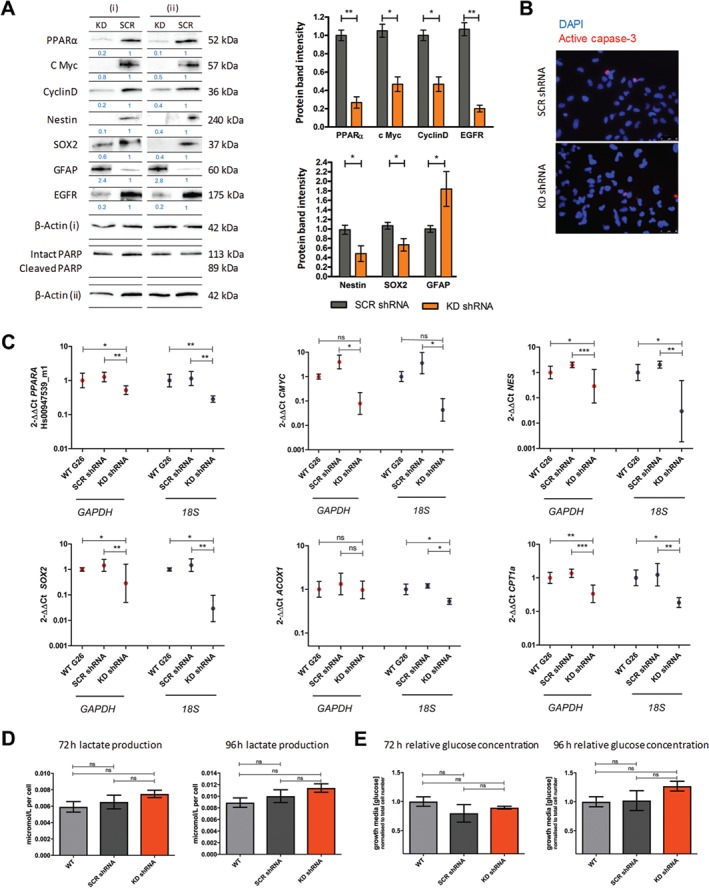
PPARα KD reduced the protein and gene expression of stemness markers *in vitro* with no effect on glycolytic flux. (A) Protein expression was examined at three independent passages, *n* = 3. Protein expression values determined using densitometric analysis, with protein‐integrated area density values expressed relative to the loading control β‐actin values. Expression values were calculated relative to the PPARα expression values. Representative western blot shown. (B) There was no significant reduction of the active caspase 3 index in the PPARα KD GSC population compared to the SCR shRNA GSC population. The proportion of active caspase 3 cellular positivity was quantified as a proportion of total nuclei per high‐power field (×200). Ten high‐power fields were examined per slide/technical replicate. Nuclei were labelled with DAPI nuclear dye. *n* = 3, three technical replicates per independent experiment. Representative active caspase 3 IF images shown. (C) mRNA expression was examined in the PPARα KD GSC population compared to the WT and SCR shRNA GSC population by RT‐qPCR, normalised to the reference genes *18S* and *GAPDH*. Relative gene expression (expressed as a fold‐difference compared to control samples) was calculated using the 2^−ΔΔCt^ method, and expression values were calculated relative to the WT control samples. The geometric mean and 95% confidence interval are shown on a logarithmic scale (to base2). *n* = 3 independent experiments; all samples analysed in triplicate. (D) Culture growth media lactate and glucose concentration was examined in three independent passages. Lactate/glucose concentrations were normalised to cell number at the time of media harvest. The concentration of analyte in blank control wells was subtracted from each assay output, which was then normalised to the total cell number in each well. The test statistic was a Wilcoxon matched pair test, two‐tailed *P* value (A) or a Friedman test with Dunn's multiple comparison test (C–E). Error bars show SEM. WT, wild type; SCR, scrambled; GAPDH, glyceraldehyde‐3‐phosphate dehydrogenase; 18S, 18 S ribosomal RNA; *p < 0.05, **p < 0.01, ***p < 0.001; ns, non‐significant.

Transduced G26 cells were examined by western blotting to assess any effects on the protein expression of key signalling mediators that occurred concomitantly with the stable KD of PPARα. The expression of c‐Myc (*p* = 0.029) and Cyclin D1 (*p* = 0.035) proteins were significantly reduced (Figure 5A). The stem cell markers nestin and SOX2 showed similarly decreased protein expression (*p* = 0.037, *p* = 0.023, respectively) (Figure 5A). The expression of the astrocytic differentiation marker GFAP was increased (*p* = 0.022) (Figure 5A). The PPARα transcription target EGFR showed a reduced protein expression (Figure 5A). Across multiple independent passages, no PARP cleavage was observed by western blot in the KD shRNA cell lines, establishing that the reduced proliferation rates described were not due to increased apoptosis. Indeed, no increase in active caspase 3 was observed by immunofluorescence in the KD shRNA cell line (Figure 5B).

Using RT‐qPCR we found a significant reduction in *PPARA* mRNA levels in the KD shRNA cell lines compared to the SCR shRNA lines when normalised to *GAPDH* (*p* = 0.022) and *18S* expression (*p* = 0.001) (Figure 5C). In keeping with the western blotting analysis of protein, there was a significant reduction in the expression of the stem cell markers *NES* and *SOX2* in the KD shRNA cell lines compared to the SCR shRNA lines when normalised to *GAPDH* (*p* = 0.001, *p* = 0.002, respectively) and *18S* expression (*p* = 0.01, *p* = 0.002, respectively) (Figure 5C). There was also a reduction in *cMYC* expression in the KD shRNA cell lines when normalised to *GAPDH* and *18S* expression (*p* = 0.025, *p* = 0.027, respectively) (Figure 5C).

The PPARα‐regulated fatty acid oxidation enzymes *ACOX1* and *CPT1a* were also examined by RT‐qPCR. A reduction in *ACOX1* was seen when normalised to *18S* expression (*p* = 0.027) (Figure 5C). There was a reduction in the expression of *CPT1A* in the KD shRNA cell lines compared to the SCR shRNA lines when normalised to *GAPDH* (*p* = 0.0002) and *18S* expression (*p* = 0.004) (Figure 5C).

### PPARα shRNA KD had no significant effect on lactate production or glucose consumption *in vitro*

Biochemical analysis was performed on media harvested from shRNA‐transduced cells after 72 and 96 h expansion *in vitro*. There was no difference in lactate production between SCR shRNA cells and KD shRNA cells after 72 or 96 h (*p* = 0.103; *p* = 0.092, respectively) (Figure 5D). There was no significant difference in relative glucose concentration in the harvested media between SCR shRNA cells and KD shRNA cells after 72 or 96 h (*p* = 0.172, *p* = 0.087, respectively) (Figure 5E).

## Discussion

A key area of investigation in the search for more effective treatments for glioblastoma is the molecular manipulation of self‐renewal and proliferation pathways in GSC 39. Direct targeting of GSC may also improve the efficacy of conventional chemo‐ and radiotherapy 40. Transcription factors overexpressed in GSC could provide effective treatment targets for novel therapeutic agents. In this study, GSC were shown to express increased levels of PPARα protein and *PPARA* transcript when compared to NSC controls. NSC share key functional and genetic similarities to GSC and are considered an ideal experimental control in this setting 30. The analyses of *PPARA* expression in accessioned microarray data cross‐validated the findings derived from our *in vitro* models. Indeed, the increased expression of *PPARA* was suggested in this work to be a significant finding shared across multiple GSC cell lines. The molecular mechanisms underlying this increased expression remain to be elucidated and are an important area of future investigation.

We selected the well‐validated IDH1‐wildtype, non‐CpG island methylated G26 GSC line as a target for our lentiviral transduction work to best recapitulate a primary glioblastoma GSC subpopulation 41. Stable KD of PPARα protein expression resulted in a significantly reduced *in vitro* growth rate. This was confirmed using flow cytometric generational tracing, which showed a decrease in the number of cell divisions per unit time. PPARα KD additionally reduced the clonogenicity of the GSC line. These results indicate that PPARα is required for, or plays a key role in, the maintenance of GSC proliferative capacity.

Examination of the PPARα KD shRNA‐transduced cells demonstrated a significant increase in senescence‐associated β‐gal staining *in vitro,* indicating the induction of senescence. Cellular senescence implies a stable and long‐term loss of proliferation capacity with no loss of cellular viability or metabolic activity 42, 43, 44. Long‐term exit from the cell cycle has been suggested as a key marker of cellular senescence 42, and PPARα KD resulted in evidence of cell cycle arrest. Morphological changes consistent with a senescent phenotype were also observed 42. It is noteworthy that this indicates that molecular senescence mechanisms may remain latently functional even in aggressive GSC populations.

A defining functional characteristic of GSC is their ability to initiate and propagate histological phenocopies of human glioblastoma when xenografted intra‐cranially in immunocompromised animals 45, 46. We used orthotopic xenotransplantation to investigate the functional requirement of PPARα to maintain the tumourigenic potential of human GSC. The xenograft brains in the control SCR shRNA experimental arm showed the key histological features of a human glioblastoma. Immunophenotyping demonstrated *IDH1* mutation‐negative tumour cells with strong nuclear ATRX expression 47, 48 and EGFR overexpression 49, 50, confirming an expression profile consistent with *IDH*‐wildtype primary glioblastoma 51. Conversely, radiological and histological examination showed that PPARα KD xenografts did not form significant tumour masses *in vivo,* indicating that GSC lacking PPARα expression have markedly reduced tumour‐initiating capacity. Nevertheless, the immunofluorescence examination of PPARα KD GSC‐engrafted brains demonstrated EGFP‐positive cells at the injection sites, confirming successful cell engraftment. We concluded that these EGFP‐positive cells have a significantly reduced proliferation rate but remain viable over an extended time course *in vivo*, in keeping with the hallmarks of senescent cells. Such scattered EGFP‐positive cells may provide sufficient signal for BLI detection in the absence of an observed tumour mass, as has been previously reported 52.

It has been shown that both PPARα pharmacological antagonism and siRNA‐mediated PPARα KD reduce the expression of c‐Myc, cyclin D1 and CDK4 in renal cell carcinoma (RCC) *in vitro* models 53. The PPARα agonist Wy‐14643 has also been shown to decrease the expression of the let‐7C miRNA in wild‐type mice, with no similar repression seen in PPARα‐null animals 54. let‐7C miRNA targets and represses c‐Myc expression 54. c‐Myc plays a role in the initiation and proliferation of glial brain tumours, and there is evidence of deregulation of the c‐Myc pathway in glioblastoma 55, 56, 57. The full transcriptional functions of c‐Myc remain to be elucidated 58, but the induction of cyclin D1 59 and the repression of p21^WAF1/CIP1^ expression have been previously reported 60, 61. We investigated a putative PPARα/c‐Myc interaction in our PPARα KD *in vitro* model: c‐Myc protein expression was found to be decreased in shRNA‐mediated PPARα KD GSC. This was accompanied by a significant decrease in cyclin D1 expression and a concomitant G1 phase cell cycle arrest.

PPARα has also been reported to play a role in EGFR phosphorylation and activation 62, 63. PPARα‐LXRα/RXRα heterodimers positively regulate *EGFR* promotor activity, and a putative PPARα DNA response element has been described upstream of the *EGFR* promoter 63. We have previously reported that *EGFR* mRNA expression significantly correlates with high *PPARA* mRNA expression in the TCGA primary glioblastoma dataset 12. In keeping with these findings in surgical tumour specimens, PPARα KD in GSC was found to significantly reduce the protein expression of EGFR *in vitro*. EGFR activation and subsequent receptor dimerisation promote cellular proliferation via activation of the MAPK and PI3K‐Akt pathways 64, and this reduction of EGFR expression may be an additional factor in the decreased expression of c‐Myc, which is an immediate early‐response gene downstream of many ligand–membrane receptor complexes 58.

**Figure 6:**
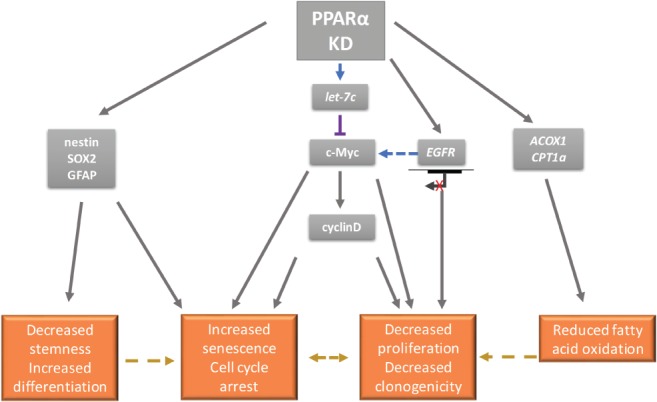
Schematic model of the effects of PPARα KD in a GSC *in vitro* model. PPARα KD exerts an anti‐proliferative effect on GSC via an altered stemness phenotype, increased rates of cellular senescence and putative changes in the metabolic flux of the GSC population. It is hypothesised that this may be attributed to downstream changes in key molecular mediators of malignant transformation such as c‐Myc, EGFR and cyclin D1.

PPARα KD also resulted in reduced expression of nestin and SOX2 proteins with an increase in GFAP protein expression. GFAP is a commonly used astrocyte maturation marker 65, 66, 67. GSC populations are known to upregulate GFAP along with other astrocyte differentiation markers (AQP4 and ALDH1A1) following the induction of a differentiated and cell cycle‐arrested state 26, 68. The altered expression of this differentiation marker was therefore in keeping with a reduction in GSC proliferative capacity and a senescent (post‐mitotic) state. Whether this PPARα KD‐driven cellular state is reversible or represents terminal differentiation warrants further investigation (Figure 6) 69.

PPARα drives the transcription of key fatty acid oxidation (FAO) enzymes, including carnitine palmitoyltransferase 1 alpha (CPT1α; *CPT1A*) and acyl‐coenzyme A oxidase 1 (ACOX1) 8. Both murine sub‐ventricular zone NSC and human GSC have been reported as being dependent on FAO 70, 71. In this study, PPARα KD reduced the gene expression of *CPT1A* and *ACOX1,* with a concomitant reduction in proliferation and clonogenic potential. PPARα antagonism in RCC models decreases FAO and enhances glycolysis 53. We assayed *in vitro* lactate and glucose concentrations and showed that a compensatory increase in glycolysis (pyruvate to lactate conversion; the Warburg effect 72) did not occur in GSC. This may be due to the reduction in c‐Myc expression, which has been associated with decreased glycolytic rates 73, 74, 75. In addition, we propose that FAO‐dependent GSC have only a small requirement for glucose oxidation 70, 76, and PPARα KD, through effects on FAO enzyme expression, may deplete GSC populations of their prime FAO bioenergetic source with no compensatory glycolytic flux, resulting in the anti‐proliferative phenotype described. Interestingly, the unique metabolic requirements of GSC compared to the aberrantly differentiated cells of the tumour mass 40 may explain the paradox of increased *PPARA* expression in mediating prolonged clinical survival 12 versus KD of PPARα in GSC inhibiting tumour growth. We hypothesise that high *PPARA* exerts an inhibitory effect on glioblastoma glycolysis 77, an effect not seen in the GSC population. The differing roles of molecular mediators of malignancy in disparate GSC and tumour mass cell populations is a key area for future investigation and has crucial implications when designing adjuvant treatment strategies to inhibit tumour recurrence.

In summary, our study establishes the expression of PPARα in GSC. The stable KD of PPARα in GSC completely abolished intracranial tumour formation. This was associated with the induction of cellular senescence *in vitro*, driven by the reduced expression of mitogenic and stemness factors. These data provide evidence of the role of PPARα in GSC as an important molecular regulator, linking proliferation and self‐renewal with a critical role in maintaining the malignant phenotype. Targeting PPARα in GSC populations may therefore have translational potential as a novel adjuvant therapeutic approach to abrogate the contribution of GSC to the poor overall clinical survival for glioblastoma patients.

## Author contributions statement

HRH, HS, CLK‐C, KCK, JU, AW, and KMK designed the study. HRH, HS, CLK‐C, TB, KMH, JR, KCK, LSB, AH, OC‐L, WGS, HB, RAK, FM, and TB performed the data collection, analysis and interpretation. HRH, HS, CLK‐C, KCK, and AW produced the manuscript. All authors approved final manuscript.

**SUPPLEMENTARY MATERIAL ONLINE**

**Supplementary materials and methods**

**Figure S1.** Western blot analysis of shRNA KD on the expression of PPARα protein

**Figure S2.** Generational tracing in KD‐ and SCR shRNA‐transduced cell lines

**Table S1.** *PPARA* shRNA primer sequences

**Table S2.** Details of primary antibodies used for western blotting

**Table S3.** Primer sets used for RT‐qPCR assays

**Table S4.** Details of primary antibodies and antigen retrieval used for immunohistochemistry

## Supporting information


**Supplementary materials and methods**

**Figure S1.** Western blot analysis of shRNA KD on the expression of PPARα protein

**Figure S2.** Generational tracing in KD‐ and SCR shRNA‐transduced cell lines

**Table S1.** *PPARA* shRNA primer sequences
**Table S2.** Details of primary antibodies used for western blotting
**Table S3.** Primer sets used for RT‐qPCR assays
**Table S4.** Details of primary antibodies and antigen retrieval used for immunohistochemistry
