# Supplementary material for: shRNA‐mediated PPARα knockdown in human glioma stem cells reduces in vitro proliferation and inhibits orthotopic xenograft tumour growth
Source: J Pathol. 2018 Dec 27;247(4):422–34. doi: 10.1002/path.5201 (PMC6462812; doi:10.1002/path.5201)
Supplement: Supplementary file 1 — Supplementary materials and methods [file PATH-247-422-s002.docx]

**shRNA-mediated PPARα knockdown in human glioma stem cells reduces *in vitro* proliferation and inhibits orthotopic xenograft tumour growth**

Haynes HR *et al*. *J Pathol* 2019 (DOI: 10.1002/path.5201)

**Supplementary materials and methods**

***Fluorescence immunocytochemistry***

Three equivalent independent passages of KD shRNA and SCR shRNA transduced cells with luciferase expression and wildtype G26 cells were plated in triplicate at 40,000 cells per well in 24-well plates (500 µl complete media per well), on sterile glass coverslips (VWR International, Lutterworth, UK) and cultured in 5% CO_2_ at 37 ^o^C for 6 days. Each well was washed 3 times in PBS, fixed for 10 min with 4% PFA and permeabilised with 100% methanol at -20 ^o^C for 10 min. Each well was washed 3 times in PBS and blocked in PBS/5% NGS/ 0.1% Triton for 30 min at room temperature. Primary antibody incubation (in blocking solution diluent) occurred overnight at 4 ^o^C. Primary antibodies used were rabbit monoclonal anti-Ki67 (1:700; Abcam, ab92742, Cambridge, UK) and rabbit monoclonal anti-active Caspase-3 (1:500; Abcam, ab32042). Each well was washed 3 times in PBS before incubation with Alexa-Fluor**®** 555 conjugated secondary goat anti-rabbit antibody (in blocking solution diluent) (1:500; Invitrogen, Carlsbad, California, USA) for 60 min at room temperature. Each well was washed 3 times in PBS and the coverslips were mounted on glass slides in Vectashield solution containing DAPI nuclear dye (H-1200, Vector Laboratories, Peterborough, UK). The cells were examined and images acquired using a fluorescence microscope (Leica DM6000B/CTR6000) and a Leica DFC350FX camera and Leica Application Suite Advanced Fluorescence software (Leica, Wetzlar, Germany). The proportion of Ki67 nuclear positivity was quantified as a proportion of total nuclei per high-power field (x200). Ten high-power fields were examined per slide. Perinuclear expression of active caspase-3 was similarly quantified.

### *Western blotting*

Cells were lysed and proteins extracted from KD shRNA and SCR shRNA transduced cells with luciferase expression, EV transduced cells and wildtype G26 cells (at 70–80% cell confluence). Protein concentration of lysates was measured and western blotting carried out. Primary antibodies used are listed in supplementary material, Table S2. Antibody specificity was validated for all antibodies. Immunoreactivity was detected using secondary IgG goat anti-rabbit/anti-mouse/anti-chicken/anti-goat HRP conjugated antibodies (1:3000; ab7090, 1:5000; ab6789, 1:5000; ab97135, 1:3000; ab97110 respectively: all Abcam) and protein expression visualised using chemiluminescence with Amersham enhanced chemiluminescent ECL Plus™ Western Blotting Detection System (1:1) (Amersham, Chicago, Illinois, USA). A Bio-Rad Universal III Bioplex imager was used to image protein expression and densitometric analysis of protein bands performed with ImageLab v5.0 software (Bio-Rad, Hercules, California, USA). β-Actin was used as a loading control.

***RT-qPCR***

RT-qPCR was performed using the StepOne Plus Real-Time PCR system and StepOne software v2.1 (Applied Biosytems, Foster City, California, USA) with Taqman® Fast Gene Expression Mastermix (Applied Biosystems) and Assay on Demand (AOD) gene expression products as listed in supplementary material, Table S3. All samples were analysed in triplicate. Relative gene expression (expressed as a fold-difference compared to control samples) was calculated using the 2^-ΔΔCt^ method relative to the reference transcripts of *GAPDH* and *18S*, and the geomean taken for each group. The amplification baseline and threshold (exponential phase of target amplification) were automatically calculated by the StepOne software. Non-template control (NTC) and RT- (gDNA contamination) control samples were included in each RT-qPCR run. *18S* and *GAPDH* AOD was used for NTC and RT- samples. Where a minimum ≤5 Ct was present between the NTC signal and sample, that sample was excluded from downstream analysis. Samples were omitted where the Ct standard deviation was ≥1.5 in the technical triplicates.

***Immunohistochemistry (IHC)***

PPARα *IHC:* Tissue sections cut at 2 µm thickness were placed on SuperFrost/Plus slides (Fisher, Waltham, Massachusetts, USA) and dried overnight at 37 ^o^C. The following stages of immunohistochemistry were performed using a Leica Bond III automated immunostainer (Leica) and a Bond Polymer Refine Detection kit (Leica). Tissue sections were deparaffinised, rehydrated and incubated with a Bond epitope retrieval solution 1 for 30 min. The slides were incubated with primary antibody (ab8934, Abcam) (1:55) for 30 min. Endogenous peroxidase activity was blocked with hydrogen peroxide for 5 min followed by poly-horseradish peroxidase (HRP) IgG secondary reagent for 15 min. The slides were then stained with 3,3’ diaminobenzidine (DAB) chromogen for 10 min, copper enhancer for 5 min and counterstained with haematoxylin for 5 min. Sections were transferred to a Leica ST5020 Multistainer and were dehydrated and cleared using standard automated procedures. Sections were then transferred to a Leica CV5030 Fully Automated Glass Coverslipper for mounting of cover slips using a standard automated procedure.

*Ki67 IHC:* Tissue sections cut at 2 µm thickness were processed using a Leica Bond III automated immunostainer (Leica) and a Bond Polymer Refine Detection kit (Leica) as above. HIER2 (pH 9.0, 20 minutes) was used followed by mouse monoclonal anti-Ki67 (K2, Leica), concentration 1 mg/l (prediluted), with standard autostainer procedures (as above). Sections were transferred to a Leica ST5020 Multistainer and were dehydrated and cleared using standard automated procedures. Sections were then transferred to a Leica CV5030 Fully Automated Glass Coverslipper for mounting of cover slips using a standard automated procedure. A positive control was included in each run of IHC along with primary antibody omission (negative reagent) controls.

*EGFR IHC*: Tissue sections cut at 2 µm thickness were processed using a Leica Bond III automated immunostainer (Leica). HIER2 (pH 9.0, 20 min) was used followed by mouse monoclonal anti-EGFR (NCL-L-EGFR Leica), concentration 1:25, with standard autostainer procedures (as above). A positive control was included in each run of IHC along with primary antibody omission (negative reagent) controls. EGFR IHC was carried out at *UCL Advanced Diagnostics*, London.

All stages of IDH1, ATRX, GFAP, p53 and synaptophysin IHC (on 2 µm tissue sections) were performed on the Ventana BenchMark ULTRA platform (Roche, Basel, Switzerland). All components used below are integrated into the BenchMark ULTRA platform. Tissue sections were deparaffinised, rehydrated and incubated with a Ventana Cell Conditioning (epitope retrieval) solution for the appropriate time period (CC1 or CC2) (see supplementary material, Table S4). Endogenous peroxidase activity was neutralised with Peroxide UV Inhibitor for 4 min. The slides were incubated with primary antibody (at stated dilution) for the appropriate time (see supplementary material, Table S4), followed by UV HRP Universal multilink (poly-HRP) secondary reagent for 8 min. The slides were then stained sequentially with UV DAB (diaminobenzidine chromogen) for 8 min, copper enhancer for 4 min and counterstained with haematoxylin for 4 min. Sections were transferred to a Leica ST5020 Multistainer and were dehydrated and cleared using the standard automated procedures. Sections were then transferred to a Leica CV5030 Fully Automated Glass Coverslipper for mounting of cover slips using a standard automated procedure. A positive control was included in each run of IHC along with primary antibody omission (negative reagent) controls.

Photomicrographs of mouse brain sections were taken using a Leica DM5500B microscope and a Leica DFC310 FX camera with Leica Application Suite software (Leica).

**FFPE immunofluorescence**

Tissue sections cut at 10 µm thickness were mounted on SuperFrost/Plus slides (Fisher), placed in Clearene (Leica) solution for 5 min, then fresh Clearene solution for a further 5 min. Sections were transferred to two changes of 100% ethanol solution for 3 min each then washed in running tap water for 10 min, washed 3 times in PBS and incubated with PBS/10% NGS/ 0.1% Triton for 60 min at room temperature. Primary antibody incubation (in blocking solution diluent) occurred overnight at 4 ^o^C. The primary antibody used was rabbit polyclonal anti-EGFP (1:500; Abcam, ab6556). Each slide was washed 3 times in PBS before incubation with Alexa-Fluor® 555 conjugated secondary goat anti-rabbit antibody (in blocking solution diluent) (1:500; Invitrogen) for 60 min at room temperature. Each slide was washed 3 times in PBS and coverslips (VWR International) were added to the slides using Vectashield solution containing DAPI nuclear dye (H-1200, Vector Laboratories). A primary antibody omission (negative reagent) control was also performed. The sections were examined and images acquired using a fluorescence microscope (Leica DM6000B/CTR6000) and a Leica DFC350FX camera with Leica Application Suite Advanced Fluorescence software (Leica).
