# Supplementary figures and images for: shRNA‐mediated PPARα knockdown in human glioma stem cells reduces in vitro proliferation and inhibits orthotopic xenograft tumour growth
Source: J Pathol. 2018 Dec 27;247(4):422–34. doi: 10.1002/path.5201 (PMC6462812; doi:10.1002/path.5201)

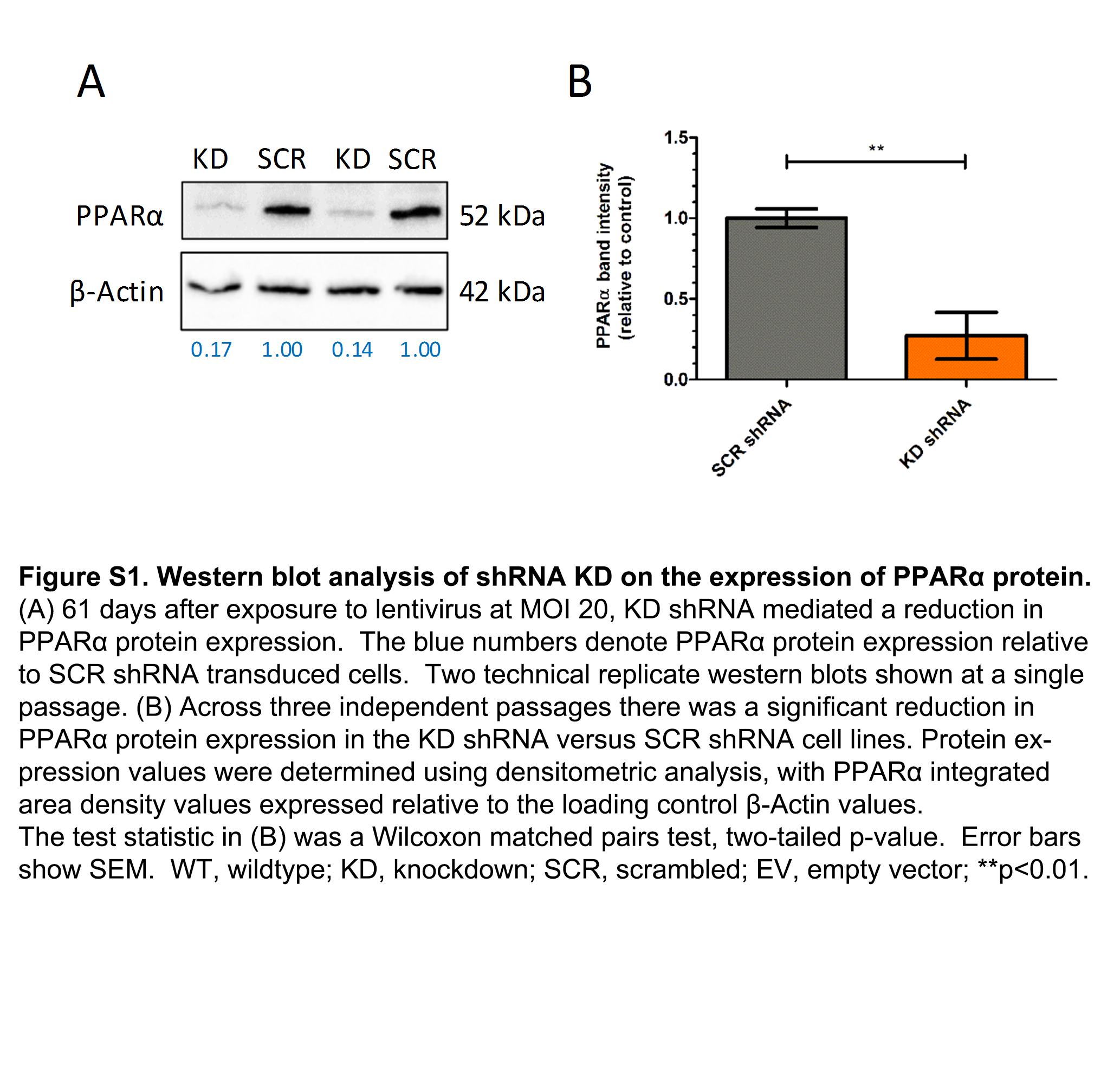

Supplement: Supplementary file 2 — Figure S1. Western blot analysis of shRNA KD on the expression of PPARα protein [file PATH-247-422-s001.tif]

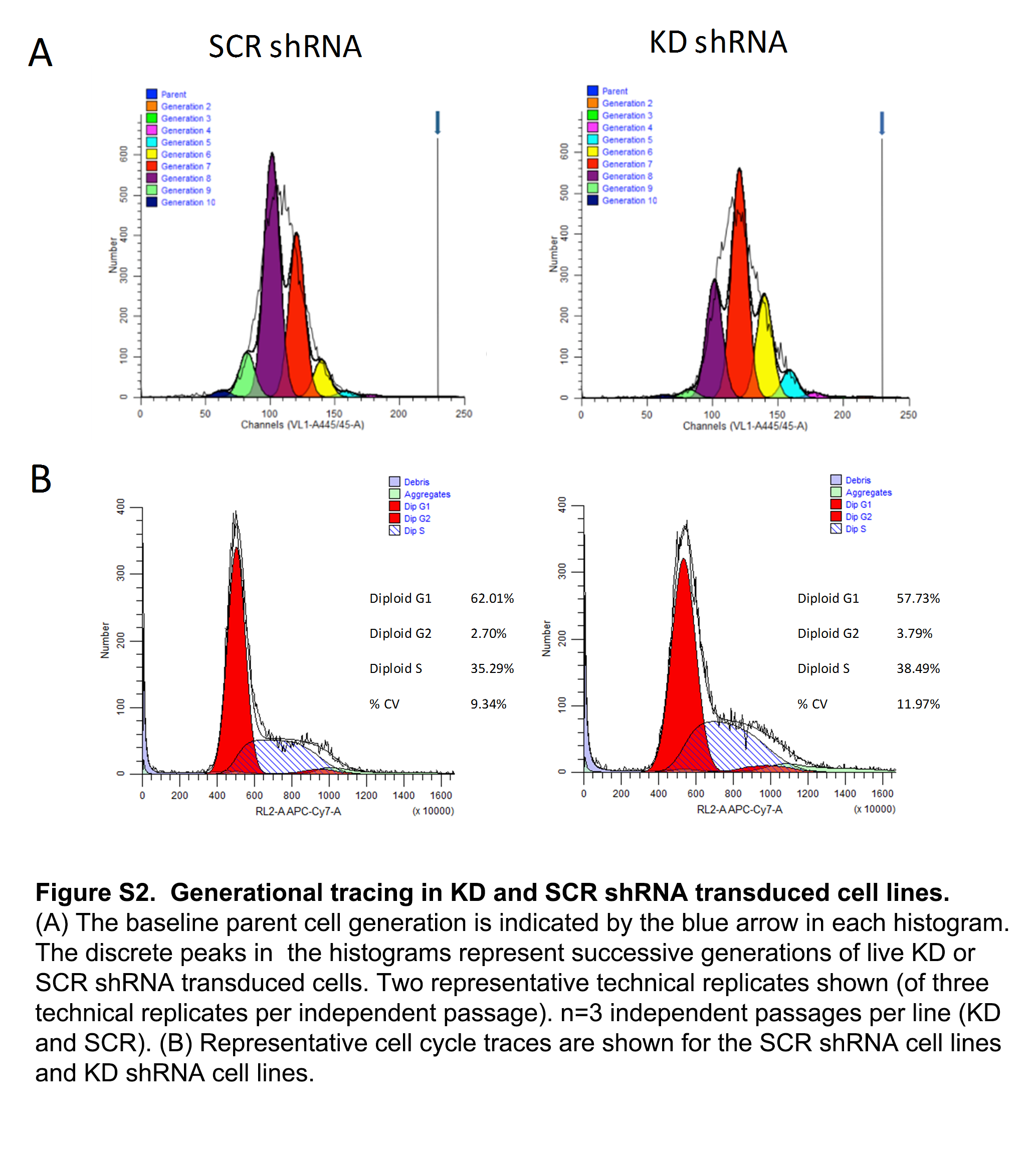

Supplement: Supplementary file 3 — Figure S2. Generational tracing in KD‐ and SCR shRNA‐transduced cell lines [file PATH-247-422-s003.tif]
