# Supplementary material for: shRNA‐mediated PPARα knockdown in human glioma stem cells reduces in vitro proliferation and inhibits orthotopic xenograft tumour growth
Source: J Pathol. 2018 Dec 27;247(4):422–34. doi: 10.1002/path.5201 (PMC6462812; doi:10.1002/path.5201)
Supplement: Supplementary file 4 — Table S1. PPARA shRNA primer sequences Table S2. Details of primary antibodies used for western blotting Table S3. Primer sets used for RT‐qPCR assays Table S4. Details of primary antibodies and antigen retrieval used for immunohistochemistry [file PATH-247-422-s004.docx]

**shRNA-mediated PPARα knockdown in human glioma stem cells reduces *in vitro* proliferation and inhibits orthotopic xenograft tumour growth**

Haynes HR *et al*. *J Pathol* 2019 (DOI: 10.1002/path.5201)


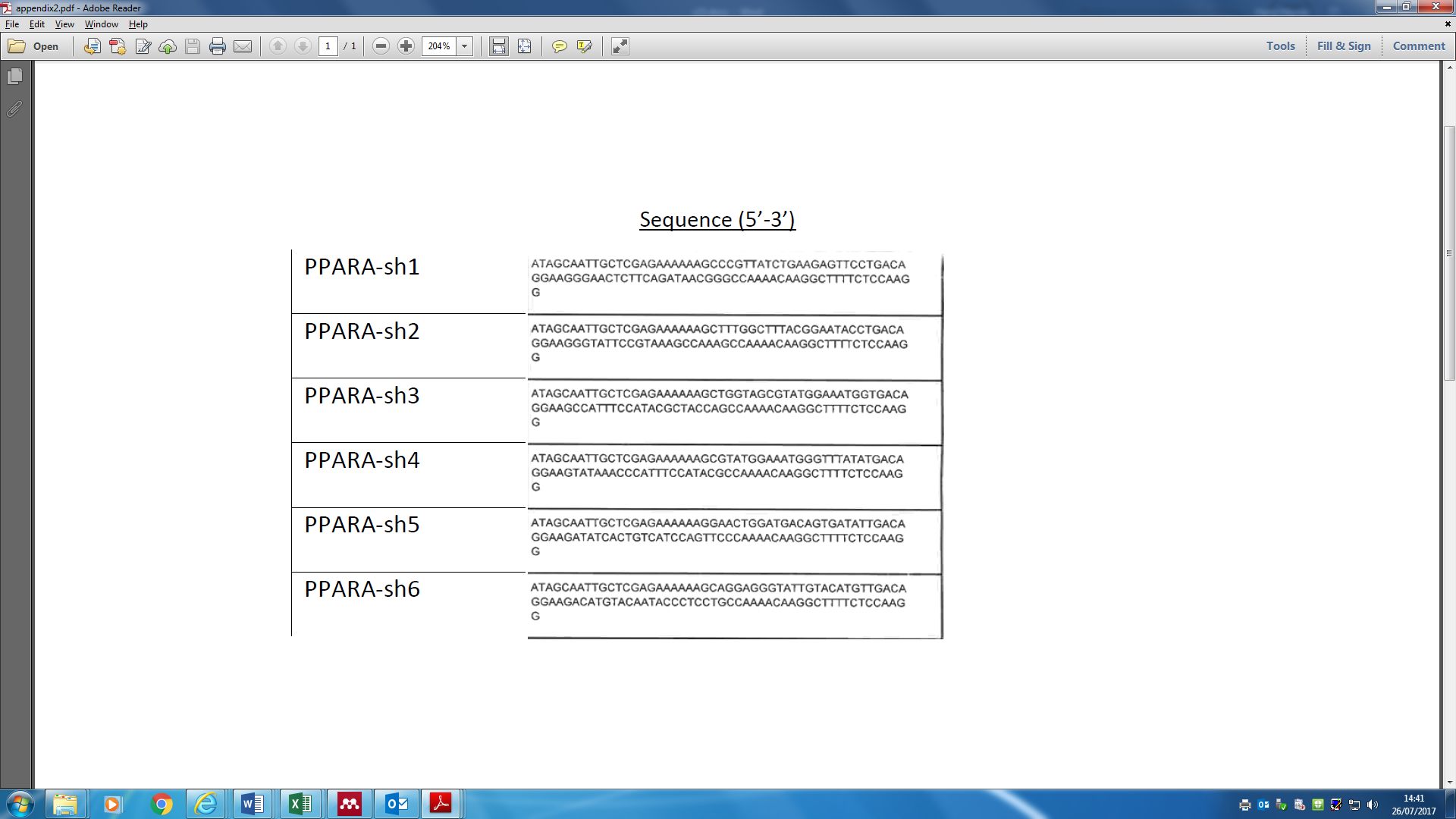


**Table S1. *PPARA* shRNA primer sequences.** Double-stranded *PPARA* shRNA fragments were produced by PCR using the above primer sequences (as supplied by Sigma, Gillingham, Dorset, UK) and a primer designed against the pSilencer plasmid (Invitrogen, Carlsbad, California, USA).

| **Target** | **Antibody (supplier’s code) clonality** | **Supplier** | **Concentration** | **Diluent**  **(in 1x TBS-T)** |
| --- | --- | --- | --- | --- |
| PPARα | (ab8934) polyclonal | Abcam, Cambridge, UK | 1:1500 | 5% MILK |
| c-Myc | (ab32072) monoclonal | Abcam | 1:1500 | 5% BSA |
| cyclin D1 | (sc8396) monoclonal | Santa Cruz, Dallas, Texas, USA | 1:500 | 5% MILK |
| nestin | (orb22944) polyclonal | Biorbyt, Cambridge, UK | 1:5000 | 5% BSA |
| SOX2 | (251176) polyclonal | ABBIOTEC, San Diego, California, USA | 1:500 | 5% BSA |
| GFAP | (AB5804) polyclonal | Millipore, Abingdon, Oxfordshire, UK | 1:1000 | 5% BSA |
| EGFR | (ab52894) monoclonal | Abcam | 1:1500 | 5% BSA |
| PARP | (556494) monoclonal | BD Pharmingen, San Jose, California, USA | 1:500 | 5% MILK |
| β-actin | (8227) polyclonal | Abcam | 1:10000 | 5% BSA |

**Table S2. Details of primary antibodies used for western blotting.**

| **Taqman® assay on demand target** | **Assay ID** | **Probes** | **Amplicon length**  **(base pairs)** |
| --- | --- | --- | --- |
| *PPARA* | Hs00947536_m1 | *FAM-MGB* | 62 |
| *PPARA* | Hs00947539_m1 | *FAM-MGB* | 120 |
| *NES* | Hs04187831_g1 | *FAM-MGB* | 58 |
| *SOX2* | Hs01053049_s1 | *FAM-MGB* | 91 |
| *CMYC* | Hs00153408_m1 | *FAM-MGB* | 107 |
| *ACOX1* | Hs01074241_m1 | *FAM-MGB* | 64 |
| *CPT1a* | Hs00912671_m1 | *FAM-MGB* | 75 |
| *GAPDH* | Hs02758991_g1 | *FAM-MGB* | 93 |
| *18S* | Hs03003631_g1 | *FAM-MGB* | 69 |

**Table S3. Primer sets used for RT-qPCR assays.**

| **Target, (Supplier’s ID) [dilution, time]** | **Supplier** | **Epitope retrieval (BenchMark ULTRA) [time]** |
| --- | --- | --- |
| IDH1R132H  (DIAH09M) [1:500, overnight] | Dianova, Hamburg, Germany | CC2 - HIER pH 6.0 [44 min] |
| ATRX  (HPA001906) [1:400, overnight] | Sigma, Gillingham, Dorset, UK | CC1 - HIER pH 9.0 [52 min] |
| GFAP  (M0761) [1:300, 60 min] | DAKO, Santa Clara, California, USA | CC1 - HIER pH 9.0 [64 min] |
| P53  (DAKO, M7001) [1:200, 48 min] | DAKO | CC1 – HIER pH 9.0 [50 min] |
| SYNAPTOPHYSIN  (DAKO, M7315) [1:50, 120 min] | DAKO | CC1 – HIER pH 9.0 [64 min] |

**Table S4.** Details of primary antibodies used for immunohistochemistry on the Ventana BenchMark ULTRA platform.
